# The WD40-protein CFAP52/WDR16 is a centrosome/basal body protein and localizes to the manchette and the flagellum in male germ cells

**DOI:** 10.1038/s41598-020-71120-9

**Published:** 2020-08-28

**Authors:** Constanza Tapia Contreras, Sigrid Hoyer-Fender

**Affiliations:** Johann-Friedrich-Blumenbach-Institute of Zoology and Anthropology – Developmental Biology, GZMB, Ernst-Caspari-Haus, Justus-Von-Liebig-Weg11, Georg-August-Universität Göttingen, 37077 Göttingen, Germany

**Keywords:** Transcription, Cytoskeleton

## Abstract

Development of spermatozoa requires remodelling and formation of particular structures. In elongating spermatids, the transient microtubular manchette contributes to the formation of the head–tail coupling apparatus (HTCA) and the sperm tail. The HTCA derives from the centrosome in that the proximal centriole inserts into the nuclear indentation and the distal centriole gives rise to the sperm flagellum. Although impairments in the formation of HTCA and sperm tail cause male infertility their molecular constituents are only partially known. The WD40-protein CFAP52 is implicated in motile cilia, but its relevance for male germ cell differentiation is not known. Here we show that CFAP52 is widespread expressed and localizes to a subset of microtubular structures. In male germ cells, CFAP52 is a component of the transient manchette and the sperm tail. However, expression of *Cfap52* is not restricted to motile cilia-bearing cells. In NIH3T3 cells, CFAP52 localizes to the centrosome, the basal body, and the mitotic spindle poles, but not to the primary cilium. Our results demonstrate that CFAP52 is not restricted to motile cilia but instead most likely functions in constituting the centrosome/basal body matrix and the sperm tail.

## Introduction

The cilia and flagella associated protein 52, CFAP52 (also named WDR16, WD repeat domain 16, WDRPUH, FLJ37528) is a member of the large WD40-repeat protein family and is widely expressed in eukaryotes. WD40-repeats, which are also known as WD or beta-transducin repeats, are short motifs of 40–60 amino acids often terminating in the dipeptide WD. The WD40-domain often comprises several of the WD40-repeats each forming a beta-pleated sheet in a propeller blade^1,2^. WD40 proteins are crucially involved in several physiological functions as diverse as signal transduction, RNA processing, remodeling the cytoskeleton, regulation of vesicular traffic, and cell cycle control^3–5^. Their immediate purpose most likely is to serve as platforms for the assembly of protein complexes and to mediate protein–protein interactions^6^.

The WD40 protein family member WDR16 is highly conserved having homologs in invertebrates and vertebrates including flies and humans^7^. In rat, expression of WDR16 was mainly observed in testis and in ependymal cells of the brain, and with reduced expression in lungs. Its expression profile, especially during ependymal development, indicated a correlation to the presence of motile cilia whereas in cells harboring primary cilia WDR16 could not be detected^7^. By immunofluorescence, WDR16 was found in the cytosol of rat testicular cells, especially in early spermatocytes and pachytene spermatocytes showing a uniform and unspecific localization. Sperm flagella stained negative for WDR16 but in Western blots the tail fraction of bull sperm revealed WDR16^7^. Overall, these results indicated that WDR16 is a marker for motile cilia-bearing cells. WDR16 depletion in zebrafish caused hydrocephalus, however, ciliary movement remained intact. Similarly, the ependymal layer did not display visible alterations^7^. WDR16, furthermore, seems to be involved in the establishment of the left–right symmetry in humans as the homozygous deletion caused laterality disorders^8^. Taken together, these data indicated, that WDR16 is functionally associated with motile cilia, either in the ependym or in the embryonic node albeit cilia seem not to be directly affected. Nevertheless, WDR16 might be involved in the stabilization of microtubular structures, as observed for the *Chlamydomonas* WDR16 ortholog FAP52 that plays an important role in axoneme stability^9^. Our intention was therefore, to elucidate the association of WDR16 with cilia in more detail by investigating its expression and localization at the subcellular level. We were interested in both motile and immotile cilia/flagella firstly taking advantage of flagella formation in mammalian spermatogenesis.

Haploid spermatids derived from the second meiotic division differentiated into functional spermatozoa in spermiogenesis. This process is characterized by reshaping of spermatids including elongation and condensation of the nucleus, and the development of specific structures as the acrosome, the transient manchette and the flagellum, and eventually the shedding of the cytoplasm^10,11^. These morphological changes produce highly polarized cells determined by the apical acrosome and the flagellum at the opposite pole. The functionality of the spermatozoon, that is propelling the sperm nucleus towards the oocyte, depends on the tight linkage between sperm tail and nucleus mediated by the head-to-tail coupling apparatus (HTCA)^10^. The HTCA and its morphological equivalent the connecting piece develops from the centrioles. During spermiogenesis, the acrosome and the paired centrioles migrate to opposite poles of the nucleus^12^. The proximal centriole inserts into the caudal area of the nucleus forming the implantation fossa whereas the distal centriole constitutes the basal body that gives rise to the axoneme^10,13,14^. The sperm flagellum, furthermore, contains additional so-called accessory structures as the outer dense fibres (ODFs) that went laterally to the microtubule (MT) doublets of the axoneme and provide elasticity and stiffness to the flagellum^15,16^.

Spermatid elongation is characterized by the formation of a transient microtubular structure, the manchette. The manchette MTs are connected to the perinuclear ring, which is closely associated to the acrosomal border, and extend progressively towards the caudal region thus forming a skirt-like structure surrounding the nucleus^17,18^. The manchette is essential for nuclear reshaping and considered as a track for the delivery of cargos to assemble HTCA and sperm tail^19,20^.

Besides being far from figured out in detail up to now, knock out mouse models were useful in shedding light on the underlying genetic causes responsible for impaired spermiogenesis and male infertility^21–23^. In this regard, several genes were identified affecting manchette formation and reshaping of the spermatid when mutated, e.g. the abnormal spermatozoon head shape (*azh)* phenotype is caused by a mutation in the *Hook1* gene^24–27^. *Hook1*-depletion, furthermore, disrupted the tight connection between flagellum and nucleus causing sperm decapitation^26^. Sperm decapitation and male infertility were also observed when the outer dense fibre protein ODF1 or one of its interacting proteins centrosomal protein coiled-coil domain containing 42 (CCDC42) or the testis-specific SUN-domain protein SPAG4L/SUN5 were mutated^28–31^. SUN-domain proteins are integral nuclear membrane proteins and are part of the linker of nucleoskeleton and cytoskeleton (LINC) complex responsible for bridging the nuclear envelope and the cytoskeleton^32^. In contrast, deficiency of SPAG4/SUN4, the second testis-specific SUN-domain protein and reported ODF1-binding protein caused disjunction of the manchette and male infertility but not decapitation^33–37^.

In order to figure out the protein interaction network responsible for HTCA and sperm tail formation we focused here on the cilia and flagella associated protein 52 (CFAP52). We used RT-PCR for expression analyses, and investigated its sub-cellular localization by immunocytology in transfected cells and in male germ cells. We found that CFAP52 is not restricted to motile cilia bearing cells but instead most likely is ubiquitously expressed. Our data indicate that CFAP52 is associated with a subset of MT structures especially centriole/centrosome derived structures, and the manchette and the axoneme in the sperm flagellum.

## Results

### Widespread expression of Cfap52

**Figure 1:**
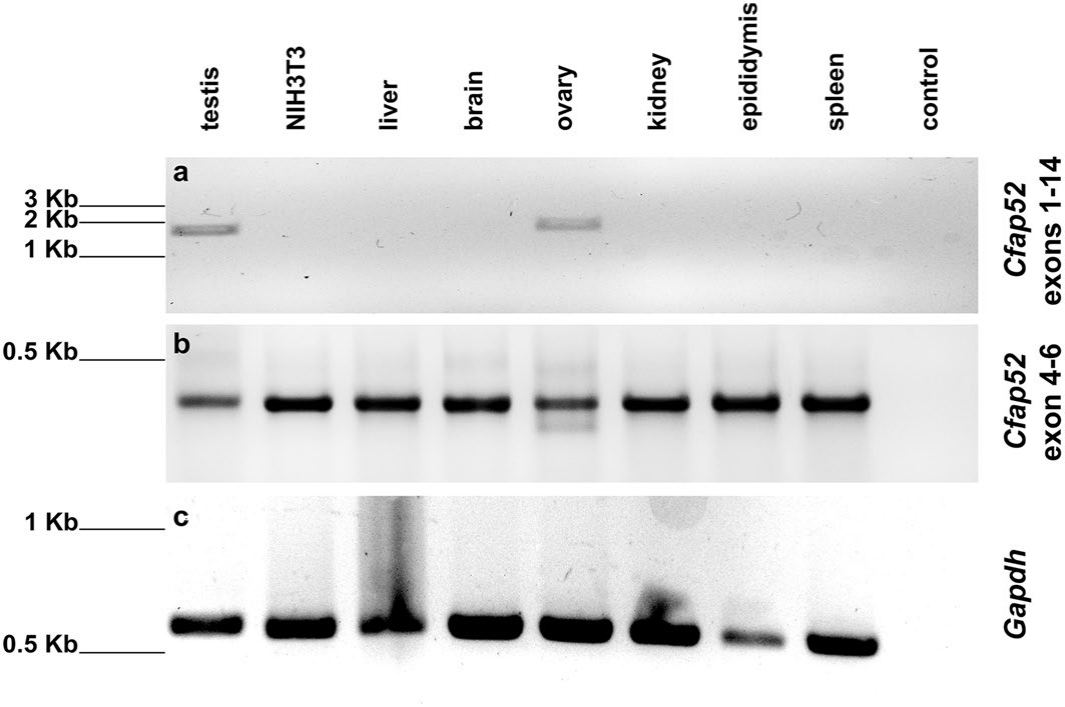
*Cfap52* expression is not correlated with the presence of motile cilia. RT-PCR on cDNA synthesized from different mouse tissues and from the fibroblast cell line NIH3T3. (**a**) Amplification of exon 1 to exon 14 of *Cfap52.* The expected fragment size of 1871 bp is found in testis and ovary. (**b**) Nested PCR on the first RT-PCR products was performed to amplify parts of exon 4 to exon 6 of *Cfap52*. The expected fragment size of 255 bp is present in all probes. (**c**) *Gapdh* amplification as quality check. Control is the PCR reaction without template.

In order to verify the reported expression of *Cfap52*, we performed RT-PCR using total RNA isolated from mouse tissues, and in addition from the mouse fibroblast cell line NIH3T3. Amplification of exons 1–14 of *Cfap52* revealed the expected fragment of 1871 bp in testis and ovary but not in any of the other probes (Fig. 1a), whereas amplification of *Gapdh* in all probes verified the quality of cDNAs (Fig. 1c). These results confirmed the previous observation of a testicular expression of *Cfap52* but additionally demonstrated that *Cfap52* is also highly expressed in ovary, which to the best of our knowledge do not harbor cells with motile cilia. However, to detect even low expression levels we performed a nested PCR on the first PCR product amplifying a region comprising parts of exons 4 to 6. A DNA fragment of the expected size of 255 bp was found in all samples (Fig. 1b). Sequencing of the PCR products obtained from brain, ovary and epididymis confirmed amplification of *Cfap52*. The different intensities obtained for the secondary PCR product are caused by differing amounts of first PCR used as template. These results demonstrate that *Cfap52* is expressed in all tissues investigated including the fibroblast cell line. *Cfap52*, therefore, seems to be ubiquitously expressed, including somatic tissues and cells that to not generate motile cilia, although at a low level. Expression of *Cfap52* is therefore not restricted to motile cilia bearing cells.

### CFPA52 localizes to the spermatid manchette and to the sperm tail

**Figure 2:**
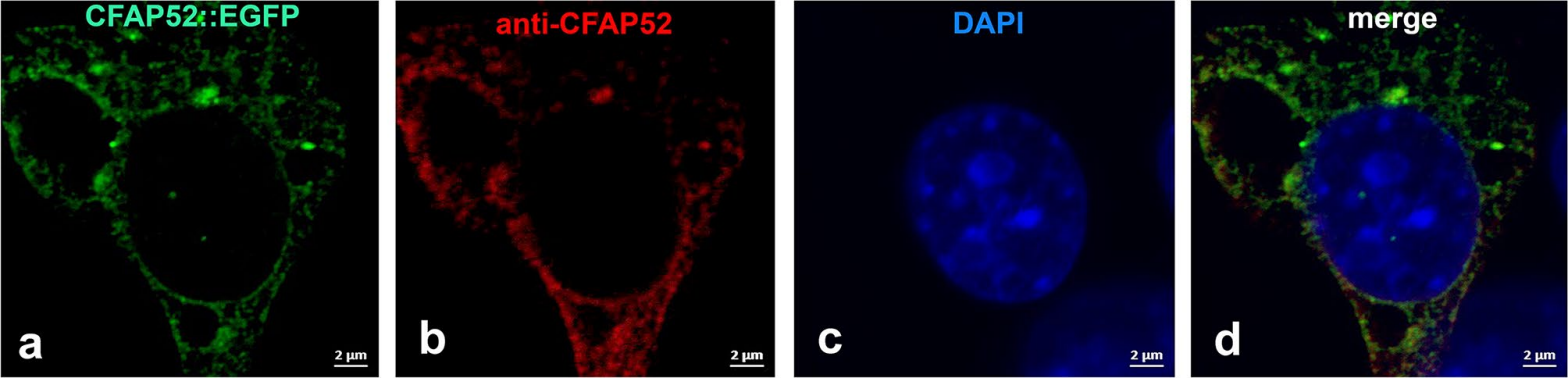
Validation of anti-CFAP52 antibody. NIH3T3 cells were transfected with the *pCfap52::Egfp* expression plasmid encoding the fusion protein CFAP52::EGFP. Proteins were detected either by their auto-fluorescence (green) or by the commercial anti-CFAP52 antibody (red). Colocalization of the inherent green fluorescence and the antibody decoration demonstrated antibody specificity (**a**–**d**). Nuclear counterstain with DAPI (blue). Bars: 2 µm.

Testicular expression of *Cfap52*, previously reported in the rat, was confirmed by our investigation in the mouse. However, besides an overall and unspecific cytoplasmic distribution of CFAP52 in spermatocytes no further specification is currently available^7^. We therefore intended to investigate the subcellular localization of CFAP52 during mouse spermatogenesis using mouse testicular cell suspensions decorated by a commercially available antibody. We first validated the anti-CFAP52 antibody for immunocytology by generating a CFAP52::EGFP fusion construct, transfected the plasmid into NIH3T3 cells, and decorated the expressed fusion protein with the anti-CFAP52 antibody (Fig. 2a–d). Antibody staining was exclusively found in transfected cells, identified by their green auto-fluorescence of the CFAP52::EGFP fusion. Furthermore, the antibody co-localized with the green fluorescent CFAP52::EGFP fusion protein.

**Figure 3:**
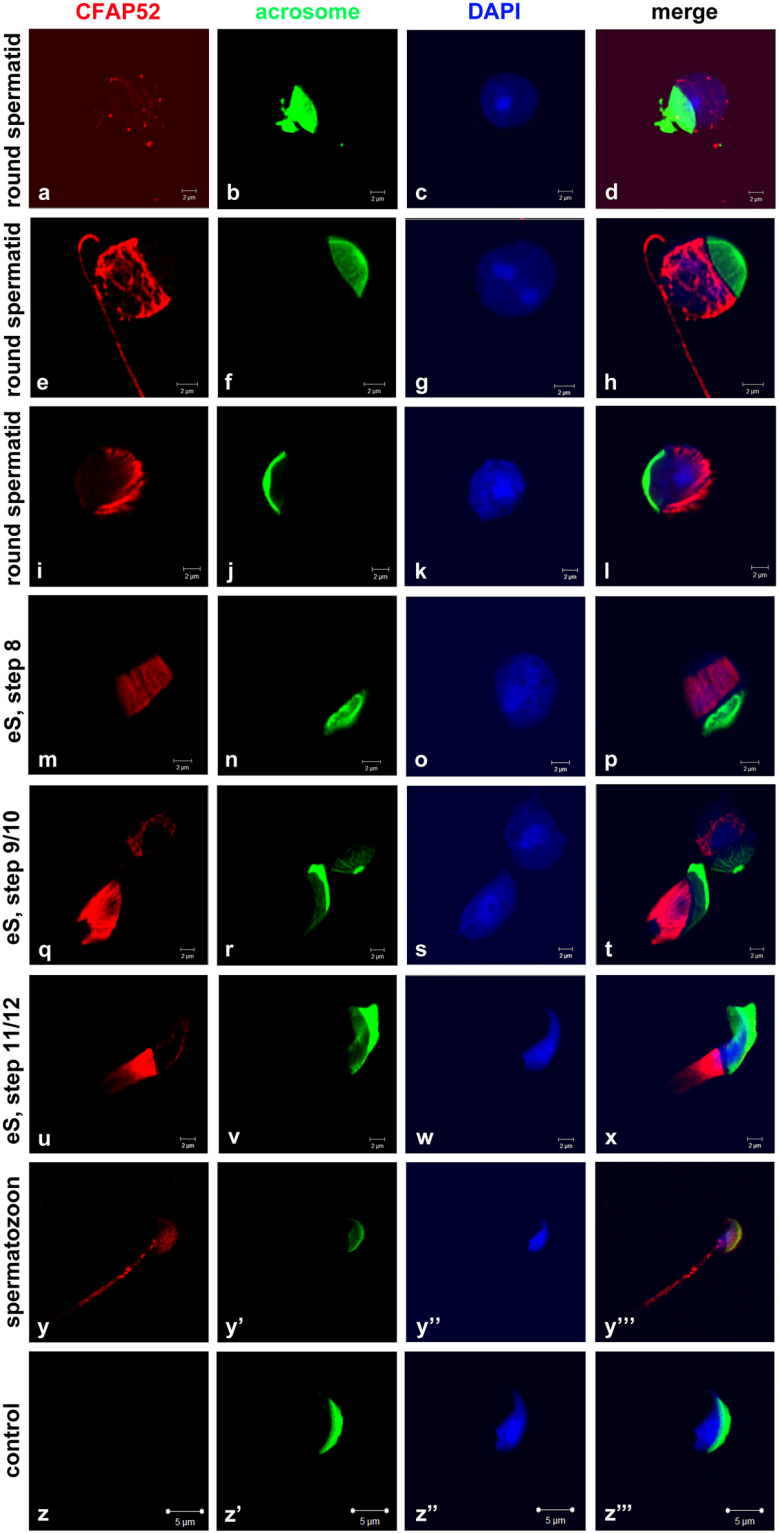
Subcellular localization of CFAP52 in male germ cells. CFAP52 is located to manchette and sperm tail in mouse male germ cells. Suspension preparations of adult mouse testis were incubated with a commercial antibody against CFAP52 (red) and the acrosome decorated with PL-FITC. The developing manchette is decorated with CFAP52 in round and elongating spermatids (**a**–**x**). In spermatozoa, CFAP52 locates to the tail with a more intense staining of the principal piece than the mid-piece (**z**–**z**″′). No staining of elongating spermatozoa was evident when the anti-CFAP52 antibody was omitted (control, **y**–**y**″′).

Having the anti-CFAP52 antibody validated, we performed immunocytology on mouse testicular cell suspensions (Fig. 3). The acrosome was decorated by peanut lectin-FITC staining to enable identification of spermatid differentiation steps (Fig. 3). A weak expression of CFAP52 with a few strong dots and a shallow indication of fibrous structures at the nuclear surroundings was first found in early round spermatids (Fig. 3a–d). These weak structures are most likely the first indications of the forming manchette, as fibre like structures with a strong CFAP52 reactivity are present in round spermatids of a more advanced step (Fig. 3e–h). Thereafter, a clear manchette with its characteristic parallel bundles was visible in round spermatids (Fig. 3i–l) and elongating spermatids (Fig. 3m–x) that is strongly stained for CFAP52 indicating an association of CFAP52 with microtubules. In mature spermatozoa, CFAP52 located to the sperm tail with a somehow stronger intensity in the principal piece than in the mid-piece (Fig. 3y–y″′). No antibody staining was detected in elongating spermatids when anti-CFAP52 antibody was omitted (Fig. 3z–z″′).

**Figure 4:**
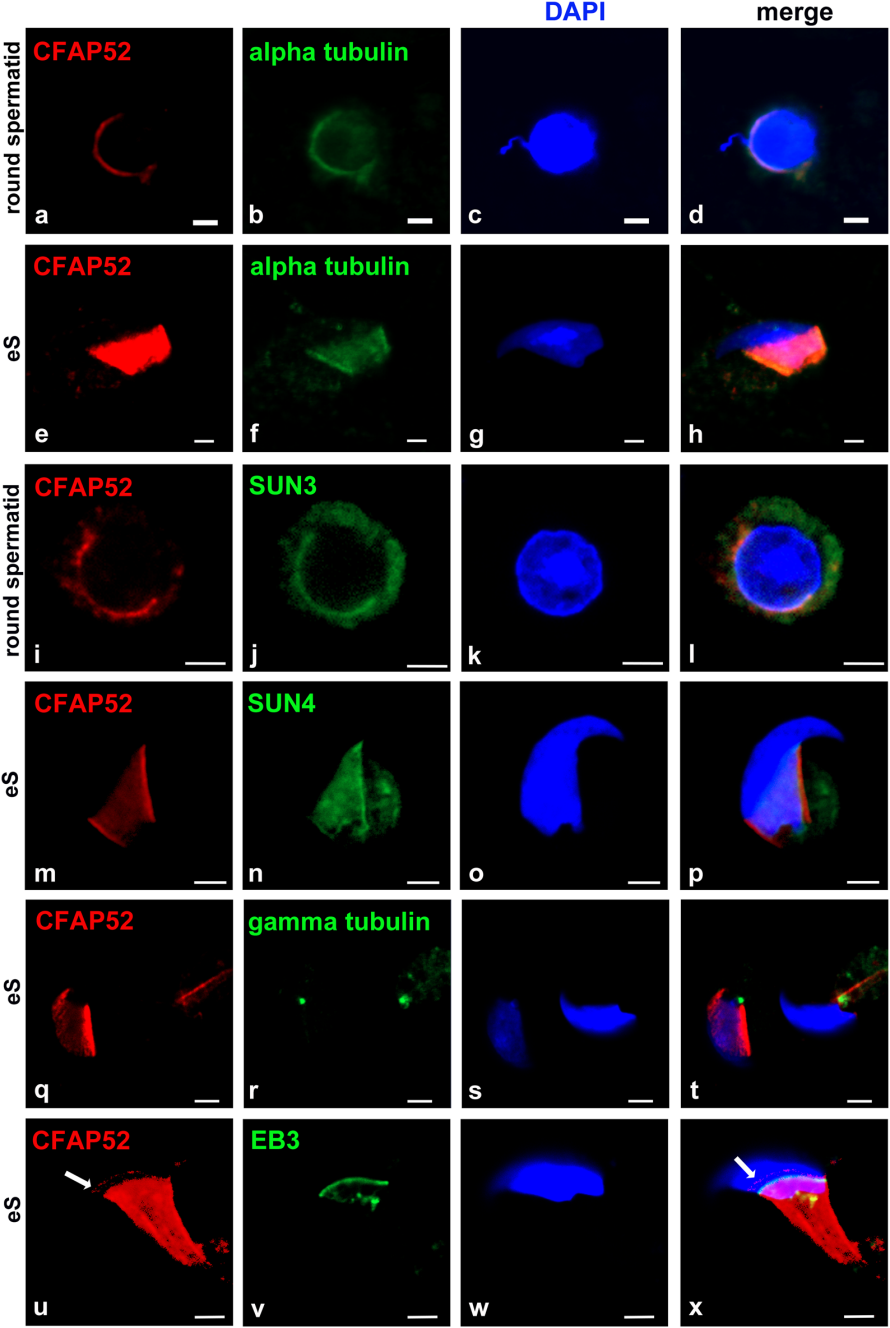
CFAP52 localizes to the manchette but not the perinuclear ring in spermatids. CFAP52 colocalizes with alpha-tubulin in the manchette in round (**a**–**d**) and elongating spermatids (**e**–**h**), and shows a similar distribution as the LINC components SUN3 (**i**–**l**) and SUN4 (**m**–**p**). Highlighting the basal body of the sperm tail by staining for gamma-tubulin could not demonstrate enrichment of CFAP52 (**q**–**t**). The microtubule plus-end tracking protein EB3 decorates the perinuclear ring that is not enriched for CFAP52 (**u**–**x**). The arrow points to the faint ring-like concentration of CFAP52 anteriorly to the perinuclear ring. CFAP52 in red (**a**, **e**, **i**, **m**, **q**, **u**), all other antibodies decorated in green (**b**, **f**, **j**, **n**, **r**, **v**). Nuclear counterstain with DAPI in blue (**c**, **g**, **k**, **o**, **s**, **w**) and merged images (**d**, **h**, **l**, **p**, **t**, **x**). eS: elongating spermatids. Bars: 2 µm (**a**–**h**) or 2.5 µm (**i**–**x**).

Colocalization of CFAP52 with the manchette microtubules was demonstrated by alpha-tubulin decoration in round and elongating spermatids (Fig. 4a–h). Furthermore, CFAP52 showed a similar distribution as the LINC components SUN3 and SUN4 (Fig. 4i–p). Enrichment of CFAP52 at the head-to-tail coupling apparatus (HTCA), which was decorated by γ-tubulin staining, was not found (Fig. 4q–t). Additionally, CFAP52 is not enriched at the perinuclear ring, which was decorated for the microtubule plus-end binding protein EB3 (Fig. 4u–x), but showed a faint ring-like concentration anteriorly to the perinuclear ring (Fig. 4u, x, arrows)^23^.

**Figure 5:**
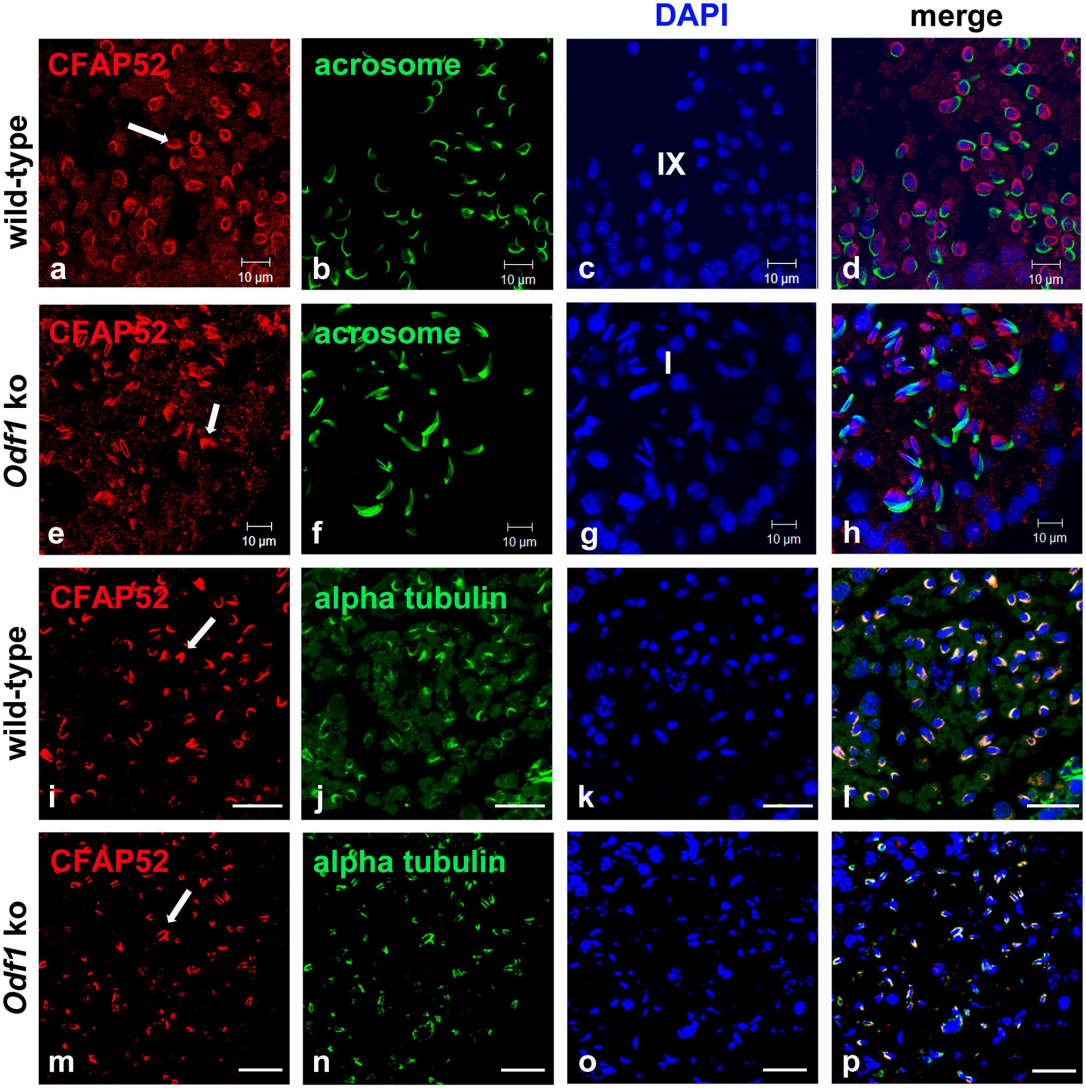
Depletion of ODF1 does not affect manchette location of CFAP52. CFAP52 decorates the manchette (arrows) in wild-type (**a**–**d**, **i**–**l**) as well as in *Odf1* ko testis sections (**e**–**h**, **m**–**p**) (in red). (**a**–**h**) Decoration of the acrosome by PL-FITC (green). Spermatogenic stages according to the acrosome formation are indicated (stage IX for the wild-type and stage I for the *Odf1* ko). (**i**–**p**) Double immunofluorescence with both, anti-CFAP52 (red) and anti-alpha-tubulin (green). Nuclear stain with DAPI (blue). (**a**, **e**, **i**, **m**) anti-CFAP52 antibody decoration (red), (**b**, **f**) PL-FITC (green), (**j**, **n**) detection of alpha-tubulin (green), (**c**, **g**, **k**, **o**) DAPI (blue), (**d**, **h**, **l**, **p**) merge. Bars: 10 µm (**a**–**h**), 20 µm (**i**–**p**).

A similar distribution in the manchette and the sperm tail was observed for the outer dense fibre protein 1, ODF1^31^, posing the question whether the observed colocalization indicates interaction or interdependency. We, therefore, investigated CFAP52 localization in testicular sections using both wild-type as well as *Odf1*-deficient mice^28^. In testis-sections of *Odf1*^+*/*+^-mice we observed CFAP52-decoration in the manchette (Fig. 5a–d, i–l, arrows). *Odf1*-deficient testis-sections exhibited the same strong CFAP52-staining in the manchette (Fig. 5e–h, m–p, arrows) demonstrated by alpha-tubulin decoration. Thus, depletion of *Odf1* neither affected the formation of the manchette nor the recruitment of CFAP52. Localization of CFAP52 to the manchette microtubules is therefore not mediated by ODF1.

### Endogenous CFAP52 localizes to centrosome/basal body, intercellular bridge and spindle poles in somatic cells

**Figure 6:**
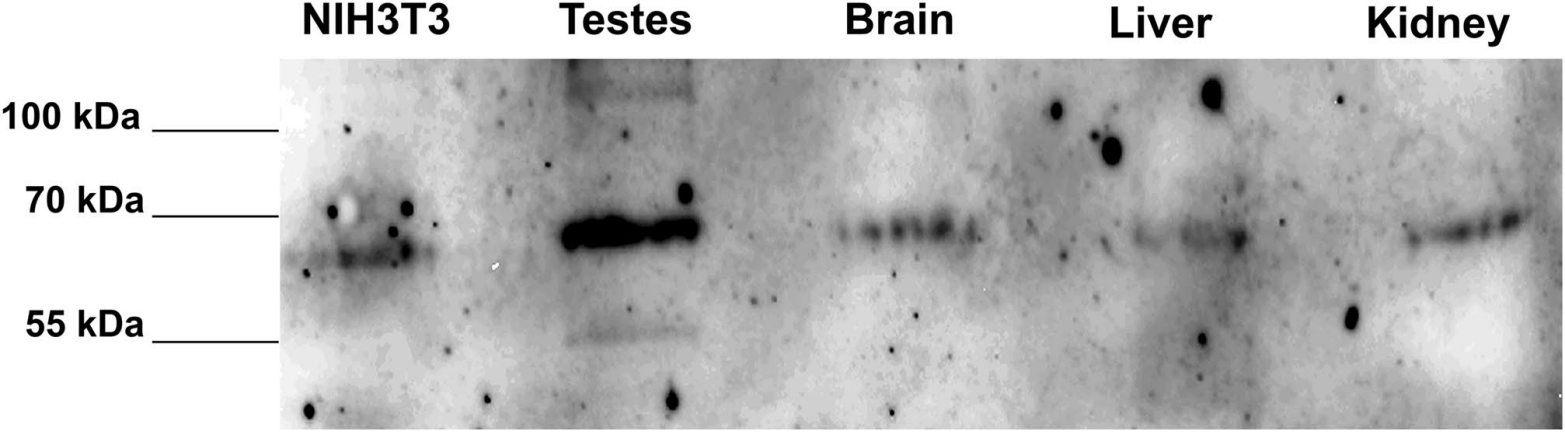
Detection of CFAP52 in mouse tissues. Total proteins of mouse tissues and of NIH3T3 cells were separated on a denaturing SDS-gel, transferred to Hybond ECL, and incubated with the anti-CFAP52 antibody. Chemiluminescence detection of the antibody**.** In all probes, CFAP52 with the predicted molecular mass of 68 kDa was detected.

**Figure 7:**
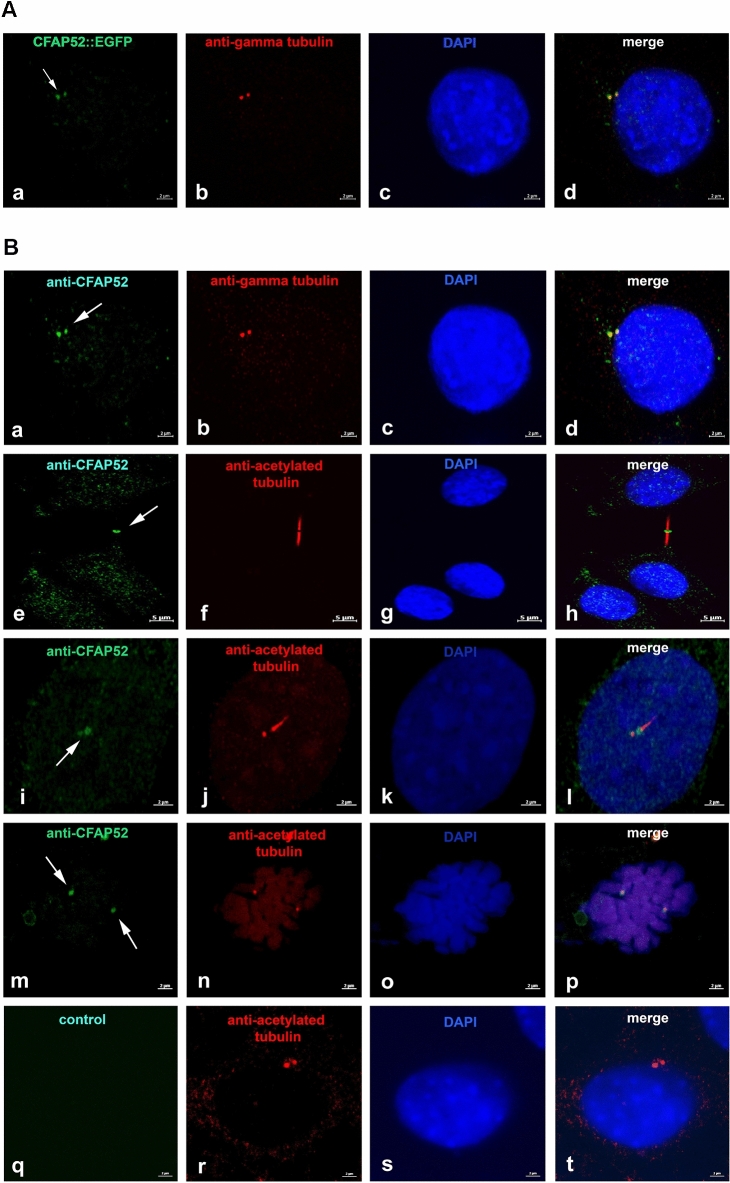
CFAP52 locates to the centrosome, the basal body, the intercellular bridge, and the spindle poles in NIH3T3 cells. (**A**) The full-length *pCfap52::Egfp* expression plasmid was transfected into NIH3T3 cells and detected by auto-fluorescence (green) (**a**). Immuno-decoration of the centrosome with the centrosomal marker γ-tubulin (red) revealed localization of CFAP52 in the centrosome (**b**–**d**). Bars are of 2 µm. (**B**) Endogenous expression of CFAP52 in NIH3T3 cells was detected by double immunostaining with anti-CFAP52 (green) and either anti-γ-tubulin (red) or anti-acetylated tubulin (red). γ-tubulin and acetylated tubulin decoration for detection of the centrosome, acetylated tubulin for decoration of the basal body, the spindle poles, the intercellular bridge, and the primary cilium. The endogenous CFAP52 (arrows) locates to the centrosome (**a**–**d**), the intercellular bridge (**e**–**h**), the basal body and its associated daughter centriole (**i**–**l**), and the spindle poles (**m**–**p**). Omitting anti-CFAP52 showed decoration of the centrosome by anti-acetylated tubulin staining (red) but no false CFAP52 staining (**q**–**t**). Nuclear counterstain with DAPI (blue). Bars are of 2 µm except for (**e**–**h**), which are of 5 µm.

We have proven prevalent low-level expression of *Cfap52* by nested RT-PCR. In order to substantiate these findings, we performed immunoblotting using total proteins obtained from mouse tissues as well as from NIH3T3 cells. CFAP52, in the expected molecular mass of ~ 68 kDa, was clearly detectable in all probes demonstrating once more that CFAP52 is not restricted to specific tissues (Fig. 6). Our results are corroborated by Western blot validation of anti-CFAP52 (www.cusabio.com). Next, we used NIH3T3 cells to isolate the coding sequence of *Cfap52* and generated a fusion protein with EGFP by cloning *Cfap52* in frame to the N-terminal end of *Egfp* in plasmid *pEgfp-N1*. The plasmid *pCfap52::egfp* was transfected into NIH3T3 cells, and its distribution analyzed. Besides a more or less uniform background fluorescence we found a specific concentration of the CFAP52::EGFP fusion protein in two closely associated spots in the vicinity of the nucleus. Immuno-decoration with anti-γ-tubulin antibodies as a centrosomal marker revealed that CFAP52::EGFP colocalized with the centrosome in NIH3T3 cells (Fig. 7A a–d). We then asked whether the centrosomal location is caused by overexpression of the fusion protein, or otherwise is a feature of the endogenous protein characterizing CFAP52 as a novel centrosomal protein. To this end, NIH3T3 cells were treated for immuno-decoration and double stained with anti-CFAP52, and either anti-γ-tubulin or anti-acetylated tubulin antibodies (Fig. 7B). We detected the endogenous CFAP52 protein in the centrosome by colocalization with the centrosomal marker protein γ-tubulin (Fig. 7B a–d). Furthermore, CFAP52 is found in the cytoplasmic bridge linking the two daughter cells (Fig. 7B e–h). In this case, the cytoplasmic bridge is highlighted by acetylated tubulin. CFAP52 did not colocalize with acetylated tubulin but instead seemed to be concentrated at the tip of the cytoplasmic bridge near the plasma membrane. NIH3T3 cells generate non-motile, solitary cilia known as primary cilia that are also easily identifiable by immuno-decoration for acetylated tubulin. The primary cilium as well as its associated daughter centriole at its base were identified by acetylated tubulin (Fig. 7B i–l). CFAP52 was not found in the ciliary axoneme but is highly concentrated at the basal body and at the daughter centriole. Additionally, CFAP52 is concentrated at the spindle poles that are highlighted by acetylated tubulin staining when captured at low intensity (Fig. 7B m–p). Omitting the anti-CFAP52 antibody and using both secondary antibodies for incubation in the controls demonstrated decoration of the centrosome with anti-acetylated tubulin antibodies (in red) but did not show any green fluorescence (Fig. 7B q–t).

### Centrosomal recruitment of CFAP52 isoforms

**Figure 8:**
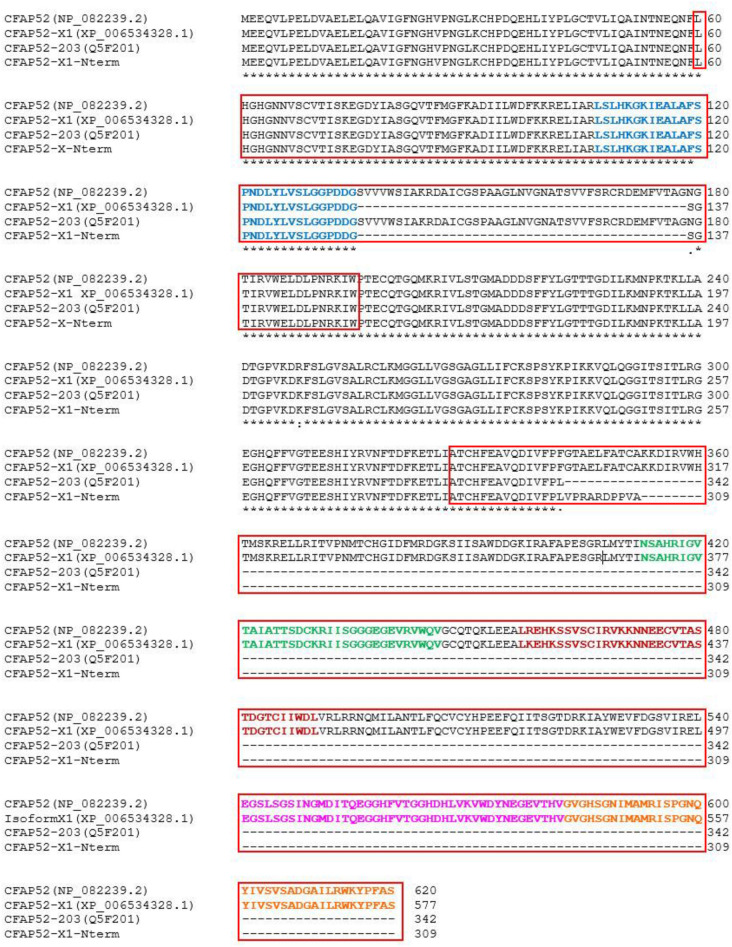
CFAP52 isoforms. *Wdr16*/*Cfap5*2 encodes three isoforms by alternative splicing. The full-length isoform CFAP52 (NP_082239.2) consists of 620 amino acids. The isoform denominated CFAP52-X1 (XP_006534328.1) consists of 577 amino acids, and the isoform CFAP52-203 (Q5F201) has a postulated length of 342 amino acids. The sequence denominated CFAP52-X1-Nterm is the N-terminal end of isoform X1 consisting of 309 aa and was artificially cloned. The WD40 repeats regions (according to Prosite circular profile) comprise aa 60 to 195 and 328 to 620 (enframed in red). The WD40 repeats (according to Prosite WD-repeats-2) are colour-coded.

**Figure 9:**
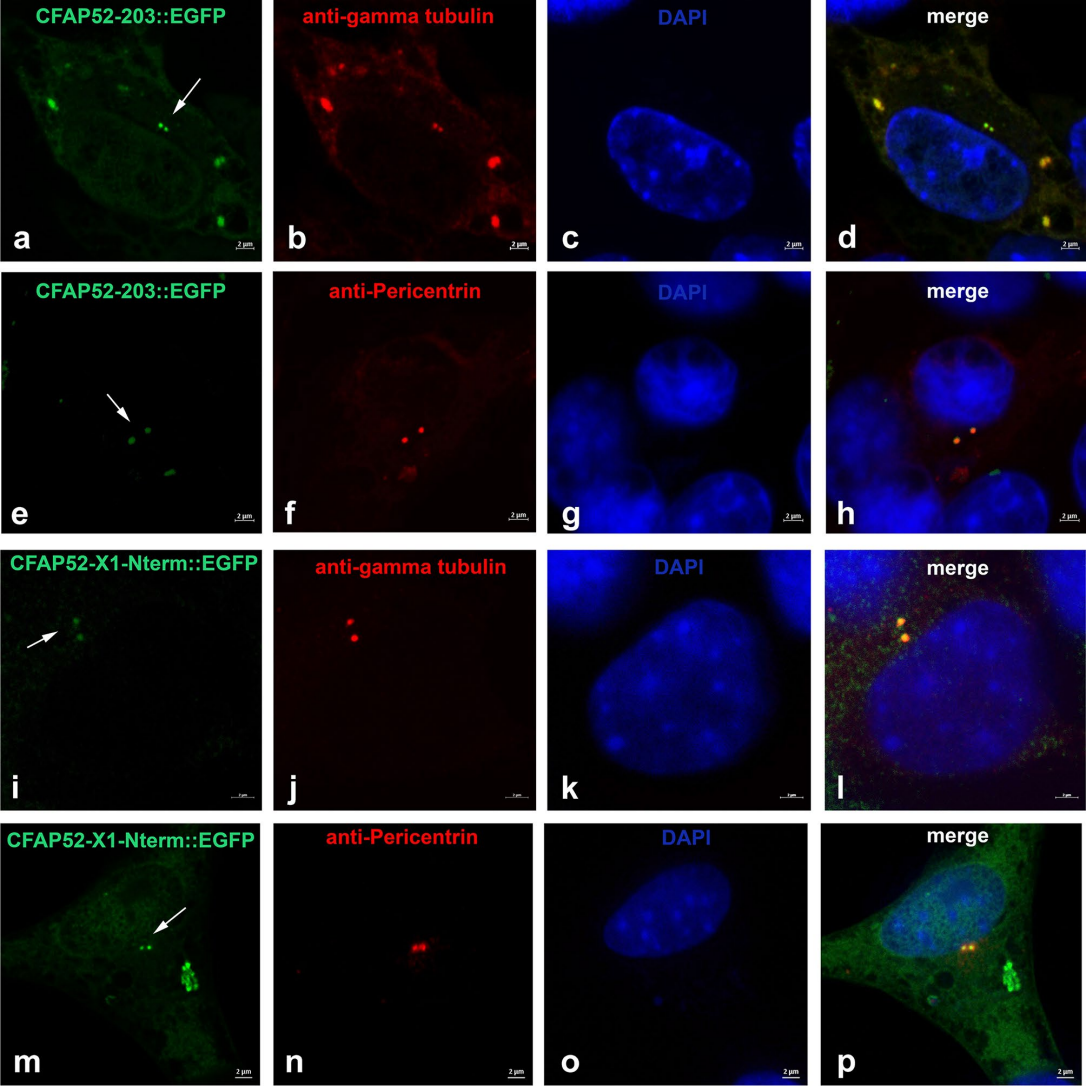
Centrosomal location of EGFP-fusion proteins of CFAP52 isoforms 203 (CFAP52-203::EGFP) and the N-terminal end of isoform X1 (CFAP52-X1-Nterm::EGFP). Expression plasmids for the isoform 203, *pCfap52-203::Egfp* (**a**–**h**), and the N-terminal sequence of CFAP52-isoform-X1, *pCfap52-X1-Nterm::Egfp* (**i**–**p**) were transfected into NIH3T3 cells and detected by the EGFP auto-fluorescence (green, **a**, **e**, **i**, **m**). Decoration of the centrosomes by immunostaining for the centrosomal marker proteins either γ-tubulin (anti-gamma-tubulin, **b**, **j**) or Pericentrin (anti-Pericentrin, **f**, **n**) (both in red). Nuclear counterstain with DAPI (blue). Arrows pointing to the centrosomal location of the fusion proteins. Bars are of 2 µm (**a**–**p**).

We have identified CFAP52 as a novel centrosomal protein in NIH3T3 cells. CFAP52 is encoded by the *Wdr16*/*Cfap5*2 gene on chromosome 11 (11B3) in mice. The gene consists of 14 exons, and encodes three isoforms by alternative splicing. The longest isoform (NP_082239.2) consists of 620 amino acids (aa) encoded by exons 1–14. The isoform denominated X1 (XP_006534328.1) consists of 577 aa by skipping of exon 4. Isoform 203 (Q5F201) has a postulated length of 342 aa and is encoded by exons 1–8. We cloned the isoforms from NIH3T3 cells and generated in fusion plasmids to *Egfp*. Additionally, we generated another fusion protein named isoform X1-Nterm that consists of 309 aa of CFAP52 by skipping exons 4 and all C-terminal exons 9–14 (Fig. 8). According to ProSite (https://prosite.expasy.org) the full-length sequences CFAP52 and CFAP52-X1 contain two WD-repeats regions comprising aa 60–195, and 328–620 (related to the full-length isoform), and five WD-repeats-2. Referring to the full-length isoform, the WD-repeats-2 sequences comprise aa 107–142, 413–446, 457–490, 541–582, and 583–620. The isoform 203 contains one WD-repeat region and one WD-repeat-2, comprising aa 107–142. The WD-region of the isoform X1-Nterm differs from that of isoform 203 by a deletion of 43 aa (Fig. 8). To investigate the impact of the WD-domains for centrosomal recruitment of CFAP52 the *egfp*-fusion plasmids were transfected into NIH3T3 cells and the fusion proteins detected by their green auto-fluorescence (Fig. 9). The centrosome was decorated by immunostaining for their marker proteins either γ-tubulin or pericentrin. As observed previously for the full-length CFAP52::EGFP fusion protein (Fig. 7A) both, the EGFP-fusion proteins of isoform 203 as well as of the N-terminal end of isoform X1 (X1-Nterm), are concentrated in the centrosome (Fig. 9).Taken together, our data indicate that (1) the C-terminal region comprising the second WD-repeats domain is not essential for centrosomal recruitment and that (2) parts of the first WD-repeats domain encoded by exon 4 are also not essential for centrosomal recruitment. Thus, a centrosomal targeting sequence, if at all present, must reside in the N-terminal end of CFAP52, represented by isoform X1-Nterm, or most likely the WD-repeats domains taken as a whole are responsible to direct CFAP52 to the centrosome.

## Discussion

WDR16/CFAP52 is a member of the WD40-protein family characterized by the WD-repeat domain. The WD40-repeats, also known as GH-WD repeats, are repetitive motifs consisting of a conserved core bracketed by GH (Gly-His) and WD, and additional sequences of variable lengths^1,38^.

The WD40-domains were first described in β subunits of transducin and other G proteins isolated from bovine tissues, and in the SCF (SKP1-CUL1-F-box protein) ubiquitin ligase complex component CDC4. WD-repeat proteins fulfill diverse biological functions including RNA synthesis/processing, signal transduction, cytoskeleton assembly, mitotic spindle formation, vesicular trafficking and cell growth^6,39^. WD-repeat domains are hence widespread and highly conserved in eukaryotes. Particularly, the WD40-repeat protein CFAP52/WDR16/WDRPUH was initially identified and found to be upregulated in human hepatocellular carcinomas. Its overexpression in NIH3T3 cells accelerated cell growth, whereas inhibition of WDRPUH/CFAP52 reduced the growth of human liver carcinoma cells and induced apoptosis^3,7^.

Previous investigation of *Cfap52* indicated that its expression correlated with the presence of motile cilia^7^. Furthermore, WDR16/CFAP52 was annotated in the Ciliome Database and was identified in mouse ciliated tissues^40^. Moreover, the *Wdr16* gene was identified as FOXJ effector gene altogether indicating a correlation of WDR16/CFAP52 with ciliation especially with the generation of motile cilia^41^. Proteins containing specific domains, especially coiled-coil domains, WD40-repeat domains, or tetratricopeptide repeat domains have all been linked to cilia^40,42,43^. In this regard, the intraflagellar transport component IFTA-1 is a WD repeat-containing protein^44^. However, WDR16/CFAP52 is not annotated in Syscilia Gold Standard (https://www.syscilia.org/goldstandard.shtml) and therefore seems not to be an essential ciliary component.

The reported association of the cilia and flagella associated protein 52 (CFAP52/WDR16) with ciliation and its high expression in testis offered the unique opportunity to investigate its sub-cellular distribution in more detail. The formation of the flagellum starts in haploid spermatids and is well known step-by-step from the migration of the paired centrioles towards the caudal end of the nucleus, their insertion into the implantation fossa, and their progressive transformation into the basal body, the connecting piece, and eventually the outgrowth of the flagellum. The proximal centriole inserts into the implantation fossa and mediates the proper linkage between head and tail by the head-to-tail coupling apparatus (HTCA). The distal centriole, on the other hand, gives rise to the basal body that in turn will seed flagellum formation^10,45^. Furthermore, flagellum and connecting piece are both discernable at light-microscopic resolution and are easily identifiable using immuno-decoration.

Formation of HTCA and sperm tail requires many proteins that are presumably transported by a transient microtubular structure, the manchette^24,27^. The HTCA is essential for functional sperm, and its malformation causes acephalic spermatozoa and male infertility due to fragile attachment of sperm head and tail or even detachment^12,46^. Albeit essential for fertilisation, the knowledge of the molecular composition of the HTCA is just in the beginning. We therefore asked, whether CFAP52 as an annotated ciliary component is associated with the flagellum or its development. We found that, opposed to previous reports, CFAP52 locates to the transient manchette in round and elongating spermatids. CFAP52 might thus be involved in the stabilization of the structure or in supporting delivery of cargos, or is a cargo protein itself. Later on, CFAP52 was found in the sperm tail with the strongest staining in the principal piece. A weaker decoration was observed in the middle piece that might be explained by a reduced antibody accessibility due to the fact that mitochondria and outer dense fibres encapsulate the central region of the axoneme. Although, location of CFAP52 to manchette microtubules and sperm tail resembles that of the major outer dense fibre protein ODF1, recruitment of CFAP52 to these structures is independent of ODF1.

Expression analyses of *Cfap52* by RT-PCR, notably nested RT-PCR, revealed transcription in all tissues investigated including those that do not harbour motile cilia, e.g. ovary. Additionally, *Cfap52* was also expressed in the fibroblast cell line NIH3T3. *Cfap52* cDNAs were isolated from NIH3T3 cells and cloned in frame to *egfp* to generate EGFP-fusion proteins when ectopically expressed. Investigations of the subcellular distributions of CFAP52::EGFP-fusion proteins as well as of the endogenous CFAP52 protein demonstrated location of CFAP52 in the centrosome, the mitotic spindle poles, the tip of the mid-bodies, and at the base of primary cilia. However, the primary cilium axoneme was not decorated by CFAP52. We observed centrosomal location of full-length CFAP52 and its isoforms including its N-terminal part comprising a single truncated WD40-domain. We propose that centrosomal recruitment most likely is mediated by the WD40-domain that generally functions in mediating protein–protein interactions^6^.

Our data indicate, that CFAP52 associates with a subset of microtubular structures, especially centrosome/centriole derived structures. As WD-repeat proteins mediate protein interactions, CFAP52 might likewise act in assembly or stabilization of protein complexes constituting the centrosomal/basal body matrix and the sperm tail^6^. Furthermore, the location of CFAP52 to the sperm tail but not to the axoneme of primary cilia suggests that CFAP52 fulfils specific functions related to the stability, maintenance, or motility of the sperm tail. A loss-of-function of CFAP52 might therefore primarily affect the sperm tail and in turn male fertility. It is currently not known whether mutations in CFAP52 affect male fertility although mutations in WD-repeat proteins, as e.g. in WDR66/CFAP251, WDR96/CFAP43, and WDR52/CFAP54, causing human male infertility due to the sperm flagella defects have been reported^47–49^. Knock down or mutation studies of CFAP52 in zebrafish and humans, respectively, indicated impaired motile cilia in the ependym causing hydrocephalus, and impaired motile, solitary cilia in the node causing left–right symmetry disorders^7,8^. However, it has not been investigated whether primary cilia and the sperm tail are also affected, causing ciliopathies and male infertility due to sperm motility disorders, respectively. We anticipate our results to be a starting point for a more sophisticated analysis of the role CFAP52 plays in the formation of centrosome-derived structures and in male germ cells using knock out approaches.

## Materials and methods

### cDNA synthesis and RT-PCR

Total RNA from adult mouse tissues (testis, liver, brain, ovary, kidney, spleen, epididymides) as well as from NIH3T3 mouse fibroblasts was prepared using peqGOLD RNApure™ (PeqLab, Erlangen, Germany) following the recommendations of the manufacturer. Total RNA was digested with Ambion® TURBO DNA-free™ DNase (Life Technologies). cDNA was synthesized using Maxima First Strand cDNA Synthesis (ThermoFisher Scientific)^31^. Detection of transcribed sequences was performed by RT-PCR using the following primer pairs: CFAP52-NheI-X1-For (GCTAGCATGGAAGAACAAGTTTTACC) and CFAP52-X1-BamHI-R (GGATCCGAAGCAAATGGGTATTTC) generating a product of 1871 bp. After first PCR, the following primers were used to perform a nested PCR: CFAP52-For (CCAGCGTGGTCTTCTCTAGG) and CFAP52-Rev (CCTTCACAGGCCCAGTATC) generating a fragment of 255 bp. Gapdh was amplified using Gapdh-For (GTATGACTCCACTCACGGCA) and Gapdh-Rev (GTCAGATCCACGACGGACAC) generating a fragment of 594 bp. PCR products were sequenced.

### Plasmid constructs

Four different *Cfap52* coding sequences were amplified by PCR. *Cfap52* full-length (NP_082239.2) and *Cfap52* isoform X1 (XP_006534328.1) were amplified using the primer pair CFAP52-NheI-X1-For (5′-GCTAGCATGGAAGAACAAGTTTTACC-3′) and CT-CFAP52-X1-BamHI-R (5′-GGATCCGAAGCAAATGGGTATTTC-3′). The isoform *Cfap52-203* (Q5F201) and the N-terminal region of the isoform X1 were amplified using the following primer pair: CFAP52-NheI-For (5′-GCTAGCCATGGAAGAACAAGTTTTAC-3′) and CFAP52-KpnI-203-Rev (5′-GGTACCAATGGAAAGACAATGTCCTGG -3′). PCR products were cloned into *pJET1.2/blunt* (ThermoFisher Scientific) followed by NheI/BamHI or NheI/KpnI digestion and subcloning into *pEGFP-N1 *(Clontech Lab., #U55762). Correct reading frames were confirmed by sequencing.

### Cell culture and immunocytochemistry

NIH3T3 cells (ATCC CRL-1658) were maintained in Dulbecco’s Modified Eagle’s Medium (DMEM), 10% (v/v) fetal bovine serum (FBS), 1000 U/ml penicillin, 1000 µg/ml streptomycin, and 20 mM l-Glutamine (all Gibco) at 37 °C and 5% CO_2_. NIH3T3 cells were grown on glass coverslips in 6-well plates and the plasmid DNA was transfected using EndoFectin™ Max Transfection Reagent, using the manufacturer´s instructions (GeneCopoeia). Twenty-four hours post transfection, cells were rinsed with phosphate-buffered saline (PBS) and fixed either in methanol for 10 min at − 20 °C or in 3.7% paraformaldehyde (PFA) for 20 min at 4 °C. Samples were then permeabilized with 0.3% Triton X-100 in PBS for 10 min at room temperature. Cells were rinsed in PBS, and non-specific binding sites were blocked by incubation in PBS containing 1% BSA and 0.3% Triton X-100 for 1 h^31^. Samples were incubated with primary antibodies: anti-CFAP52 (CSB-PA839781LA01HU; Wuhan Huamei Biotech Co., Ltd., Wuhan, China, Cusabio), anti-Pericentrin (PRB432C, Covance), anti-acetylated tubulin (6-11B-1; Sigma-Aldrich), anti-γ tubulin (GTU-88, Sigma-Aldrich) at 37 °C for 1 h. Secondary antibodies used are goat anti-mouse-IgG DyLight 488 (#35503, ThermoScientific), and goat anti-rabbit-MFP590 (#MFP-A1037, Mobitec). DNA was counterstained with DAPI. Images were taken by confocal microscopy (LSM 780, Zeiss) and processed using Adobe Photoshop 7.0.

### Immunocytology on testicular cell suspensions

Fresh testes from laboratory mice of strain C57/Bl6 were minced in PBS containing 2% paraformaldehyde, 0.02% SDS, and 0.15% Triton X-100, and transferred onto superfrost slides. Cells were blocked for 1 h in PBS containing 1% BSA and 0.3% Triton X-100. Anti-CFAP52 (CSB-PA839781LA01HU; Wuhan Huamei Biotech Co., Ltd., Wuhan, China, Cusabio) was used as 1:100 dilution and incubated at 4 °C overnight. CFAP52 was detected using the secondary antibody goat anti-rabbit-IgG (H + L) Alexa Fluor R 555 (F[ab]2 fragment; #A21430, Life Technologies) used as 1:1,000 dilution. Additionally, the following first antibodies were used: anti-α-tubulin (DM1A, Calbiochem, #CP06), anti-γ-tubulin (GTU-88, Sigma-Aldrich, #T6557), anti-EB3 (EB3(7), Santa Cruz, sc-136405), anti-SUN3 and anti-SUN4^36,50^, and detected using goat anti-mouse-IgG DyLight488 (Thermo Scientific, #35503) or goat anti-guinea pig-IgG Alexa Fluor 488 (Life Technologies, #A11073). DNA was counterstained with DAPI (4′,6-Diamidino-2-phenylindole; Sigma D-9542), and the acrosome was decorated with FITC-labelled peanut lectin (PL-FITC). Images were taken by confocal microscopy (LSM 510, Zeiss) and processed using Adobe Photoshop 7.0.

### Histology

Testes were fixed in 4% paraformaldehyde in phosphate-buffered saline (145 mM NaCl, 7 mM Na_2_HPO_4_, and 3 mM NaH_2_PO_4_; PBS) and embedded in paraffin. 4 µm sections were cut and placed onto Superfrost slides. After deparaffinization and rehydration probes were treated for enzymatic epitope retrieval by incubation with 0.5% Trypsin for 10–20 min at 37 °C. Specimen were blocked in PBS containing 1% BSA and 0.3% Triton X-100 followed by antibody incubation with rabbit anti-CFAP52 (CSB-PA839781LA01HU) at 4 °C overnight. Anti-CFAP52 was detected with goat anti-rabbit-MFP590 (#MFP-A1037, Mobitec) (diluted 1:100). The acrosome was stained with FITC-labelled peanut-lectin (PL-FITC) and the DNA by DAPI (4′, 6-Diamidino-2-phenylindole; Sigma D-9542).

### Western blotting

Proteins were prepared from mice tissues (testes, brain, liver, kidney) and NIH3T3 cells. Tissues were homogenized with a Dounce homogenizer in lysis buffer [150 mM NaCl, 1% Nonidet P40,0.5% sodium deoxycholate, 0.1% SDS, 50 mM Tris–HCl, pH 7.6, protease inhibitor cocktail (Halt Protease Inhibitor Cocktail 100X, Thermo Fisher Scientific, #78438)]. NIH3T3 cells were harvested by trypsinization followed by rinsing twice in phosphate-buffered saline (PBS). NIH3T3 cell pellet was resuspended in lysis buffer. Protein lysates were sonicated 3 times for 45 s each. Total protein lysates were resuspended in 2 × SDS-sample buffer and heated to 95 °C for 10 min. Proteins were separated on SDS-PAGE and transferred onto nitrocellulose membrane (Amersham Hybond-ECL, GE Healthcare)^51,52^. The membrane was blocked in 5% dry milk in TBST (10 mM Tris–HCl pH 7.6, 150 mM NaCl, 0.05% Tween20) for one hour. Afterwards, the membrane was incubated with the primary antibody rabbit anti-CFAP52 (CSB-PA839781LA01HU) in blocking solution at 4 °C overnight. First antibodies were detected by incubation with HRP-conjugated secondary antibody goat anti-rabbit IgG (Jackson ImmunoResearch, WestGrove, PA, USA). Signals were detected using ClarityMax Western ECL Substrate (Bio-Rad, #1705062) and images were captured with Chemdoc (Bio-Rad).

### Ethics statement

All mouse experiments were reviewed and approved by the animal welfare commission of the University Medical Faculty and Niedersächsisches Landesamt für Verbraucherschutz und Lebensmittelsicherheit. Licence for animal experiments has been obtained by the Institute of Human Genetics. The guidelines of the German Animal Welfare Act (German Ministry of Agriculture, Health and Economic Cooperation) were strictly followed in all aspects of mouse work. *Odf1*-ko mice has been previously described^28^.

## Supplementary information

Supplementary file1

## Data Availability

All data generated or analysed during this study are included in this published article.
