# Supplementary material for: The WD40-protein CFAP52/WDR16 is a centrosome/basal body protein and localizes to the manchette and the flagellum in male germ cells
Source: Sci Rep. 2020 Aug 28;10:14240. doi: 10.1038/s41598-020-71120-9 (PMC7455747; doi:10.1038/s41598-020-71120-9)

**Supplementary Information**

# The WD40-protein CFAP52/WDR16 is a centrosome/basal body protein and localizes to the manchette and the flagellum in male germ cells

Constanza Tapia Contreras and Sigrid Hoyer-Fender

## Supplemental Figure S1

**Original Western blot of Fig. 6.**

**Detection of CFAP52 in mouse tissues.**

Total proteins of mouse tissues and of NIH3T3 cells were separated on a denaturing SDS-gel, transferred to Hybond ECL, and incubated with the α-CFAP52 antibody. Chemiluminescence detection of the antibody**.** In all probes CFAP52 with the predicted molecular mass of 68 kDa was detected. CFAP52 is highly expressed in testis, whereas in NIH3T3 cells, brain, liver, and kidney tissues CFAP52 expression is weaker. The protein detected above the 180 kDa mass ruler most likely reflects insoluble aggregates.

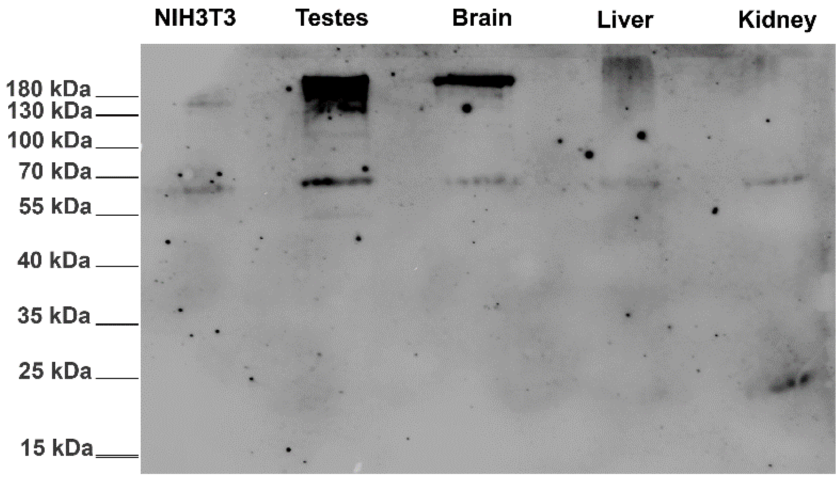

Supplement: Supplementary file 1 — Supplementary file1 [file 41598_2020_71120_MOESM1_ESM.pdf]
